# Dynamic biomechanical equilibrium in pelvic organ prolapse: from mechanistic insights to precision reconstruction

**DOI:** 10.3389/fmed.2025.1637133

**Published:** 2025-10-08

**Authors:** Daoming Tian, Qian Luo, Xingqi Wang, Yubin Wen, Yuan Li, Jiangna Gu, Hongcheng Li, Jihong Shen, Ling Li

**Affiliations:** Department of Urology, Kunming Medical University First Afiliated Hospital, Kunming, China

**Keywords:** pelvic organ prolapse, biomechanics, levator plate, posterior fornix, reconstructive surgery

## Abstract

**Background:**

The traditional treatment of pelvic floor organ prolapse (POP) is based on static anatomical repair, but the postoperative recurrence rate is still high in the surgical rate, which suggests the need to re-examine its pathogenesis from a biomechanical perspective.

**Objective:**

To propose a new concept of POP prevention and treatment centered on the dynamic mechanical balance system of the levator plate-perineum and posterior vaginal vault, and to provide a theoretical basis for clinical intervention.

**Results:**

This study reveals the key mechanisms of the pelvic floor dynamic balance system through biomechanical analysis. The stability of the pelvic floor is maintained by three synergistic aspects: first, the triangular support structure composed of the bladder-uterus-tibial plate realizes effective stress transmission; second, the posterior fornix of the vagina serves as a mechanical fulcrum, guiding the uterus to produce the characteristic “downward-backward” displacement; and lastly, the 90° functional folding angle of the vagina ensures the reasonable distribution of the loads. When this sophisticated system becomes unbalanced due to birth injury or aging, it leads to abnormal stress transmission and organ displacement, ultimately leading to prolapse symptoms.

**Conclusion:**

Shifting from static repair to dynamic mechanical balance reconstruction is the key to improving POP efficacy, and individualized mechanical repair strategies and long-term maintenance mechanisms need to be further explored in the future.

## Introduction

Pelvic organ prolapse (POP), a prevalent manifestation of pelvic floor dysfunction, represents a significant public health burden disproportionately affecting postmenopausal women. Epidemiological studies estimate that 30–50% of parous women experience POP symptoms, with 12–19% progressing to surgical intervention (1). Conventional pathophysiological models emphasize static anatomical defects - particularly ligamentous laxity and muscular avulsions (2) informing current surgical approaches like anterior/posterior colporrhaphy and sacrocolpopexy (3). However, persistent recurrence rates [12–23% post-repair (4)] challenge this paradigm, suggesting fundamental gaps in our understanding of pelvic support mechanisms.

Emerging biomechanical evidence necessitates reconceptualizing POP as a dynamic equilibrium disorder rather than a static structural failure. This is consistent with the fact that DeLancey’s team’s research has shifted from static anatomical descriptions to modeling dynamic biomechanical systems (5). This paradigm shift identifies three interdependent systems maintaining pelvic stability: (1) force-coupling between the levator plate and perineal body, (2) the posterior fornix functioning as a biomechanical pivot, and (3) coordinated neuromuscular regulation. Disruption of this tripartite system—whether through obstetric trauma, age-related degeneration, or neuromuscular dysfunction—precipitates characteristic prolapse patterns through altered force transmission vectors and loss of apical support integrity. The theoretical hypothesis was validated by experimental data in 20 cases of biomechanical analyses that have been completed by our team in the previous period (Table 1).

**Table 1:** Key components and biomechanical mechanisms of pelvic floor stability.

Component	Mechanism of action	Consequence of failure
Levator plate-perineal body complex	Generates supero-anterior force to close the hiatus	Hiatal widening, impaired closure
Posterior fornix	Acts as a fulcrum to redirect forces posteriorly	Altered displacement vectors, apical descent
Neuromuscular control	Coordinates reflexive muscle contraction	Delayed or weak response to stress

## Hypothesis

### Integrated analysis of dynamic mechanical equilibrium mechanisms for pelvic organ stability

**Figure 1:**
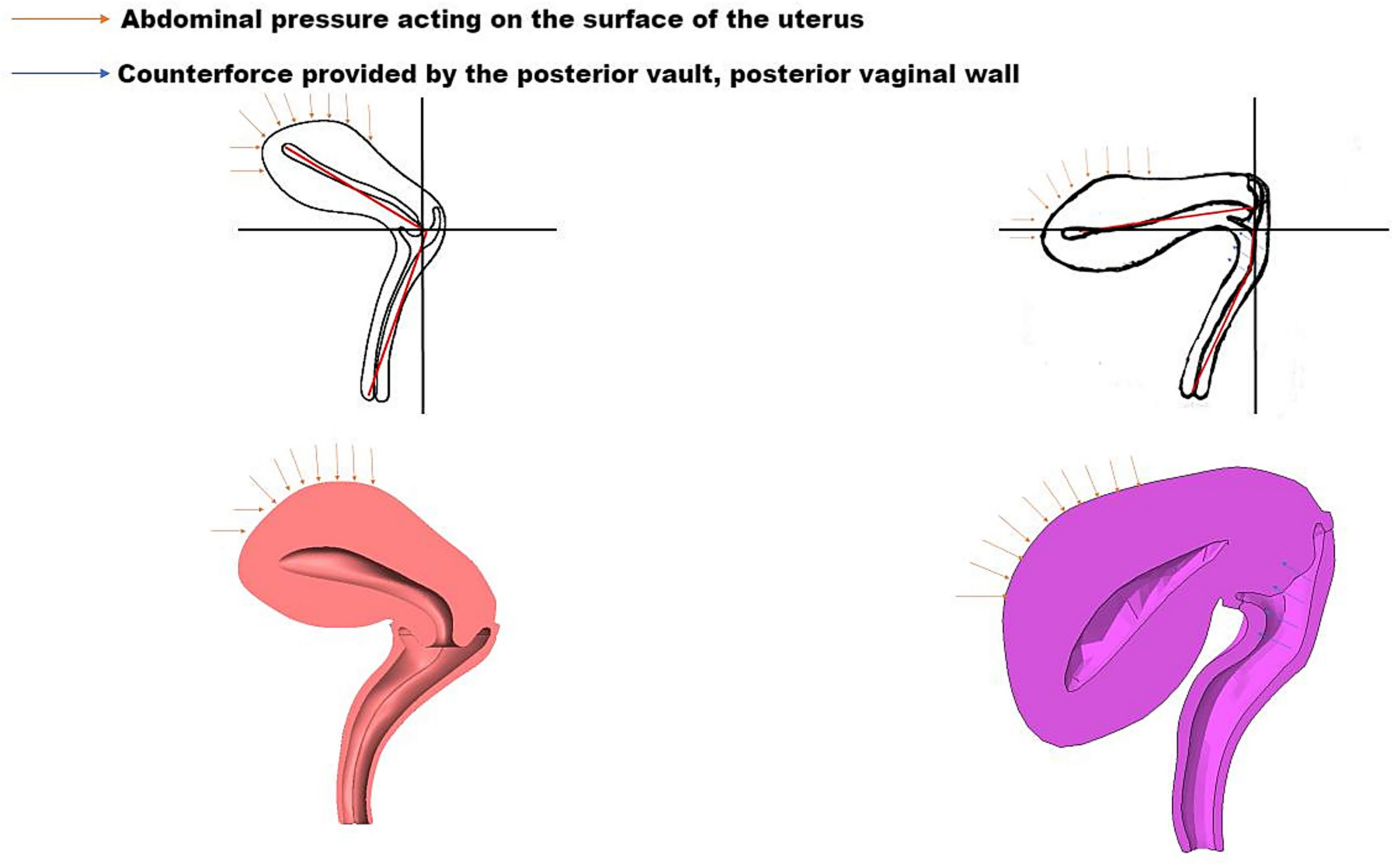
Biomechanical equilibrium of the bladder-uterus-levator plate triangular support structure under compression.

**Figure 2:**
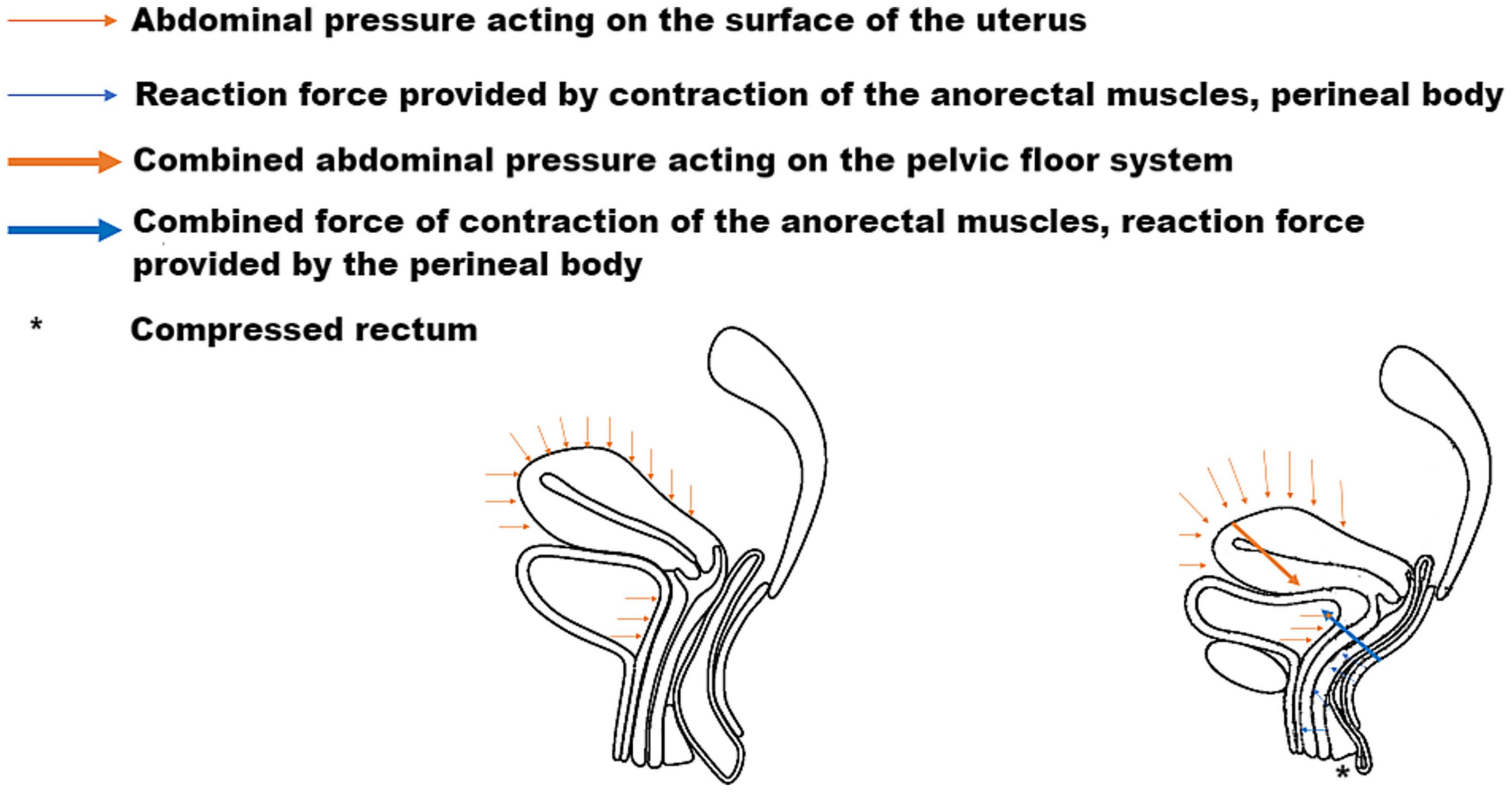
Dynamic equilibrium of the pelvic floor in response to abdominal pressure, demonstrating force vectors and muscular compensation.

The pelvic floor system maintains stability through a dynamic equilibrium mechanism involving three integrated components. Anatomically, the highly compliant bladder transmits stresses primarily to the levator plate-perineal body complex during filling, due to its mobile apex and firm posterior vaginal attachment (6, 7). Concurrently, the anteverted-flexed uterine position creates an acute vaginal-uterine angle that decreases during increased abdominal pressure, aligning with the upper vaginal segment (8). Dynamic imaging and computational modeling (9) demonstrate that abdominal pressure induces primarily posterior-inferior uterine displacement, while coordinated levator plate contraction generates counteracting supero-anterior forces. This interaction establishes a characteristic biomechanical equilibrium featuring: (1) parallel vaginal-uterine alignment, (2) a 90° vaginal-levator plate angle, and (3) a stable bladder-uterus-levator plate triangular structure. As illustrated in Figure 1, this configuration facilitates efficient stress redistribution toward the sacrococcygeal axis during sudden pressure increments (10). The integrity of this system relies critically on levator plate morphology and neuromuscular coordination—any disruption may compromise this self-stabilizing mechanism, as further demonstrated in Figure 2 under simulated abdominal pressure.

### Biomechanical mechanisms of the posterior vaginal vault

The posterior fornix, though historically underappreciated, serves as a critical biomechanical stabilizer in pelvic floor function (11). Its unique anatomical architecture—characterized by differential wall lengths (6–7 cm anteriorly versus 9–11 cm posteriorly) and strategic positioning between the uterine cervix and rectal ampulla—forms a sophisticated load-bearing mechanism (12). During normal function, apposition of the vaginal walls creates a closed lumen that efficiently transmits and distributes mechanical stresses (13). Under increased abdominal pressure, maintenance of the 90° vaginal angulation redirects axial stresses toward the sacrococcygeal axis rather than the vaginal introitus.

**Figure 3:**
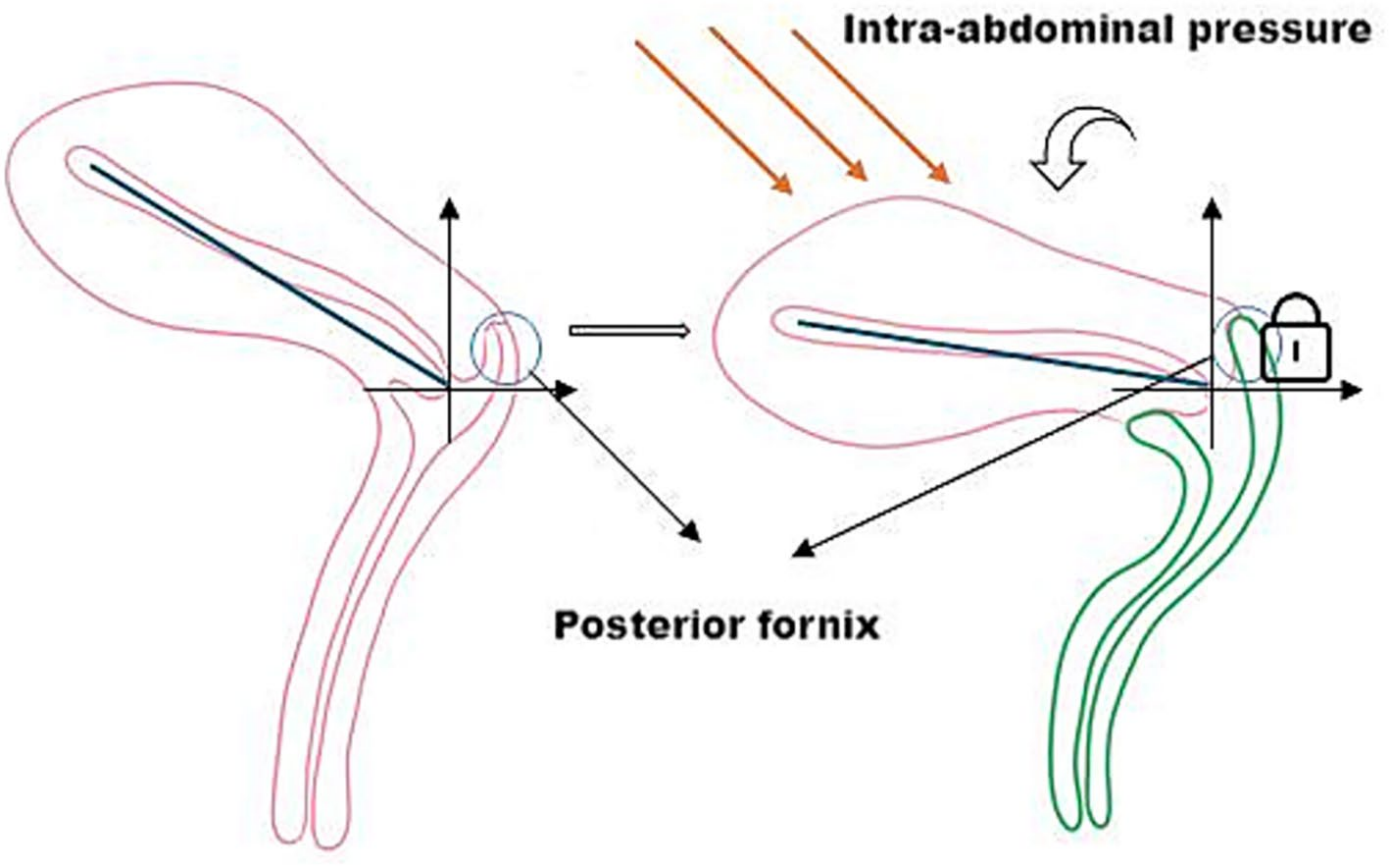
The posterior fornix as a biomechanical pivot guiding posteroinferior uterine displacement and stress redirection.

**Figure 4:**
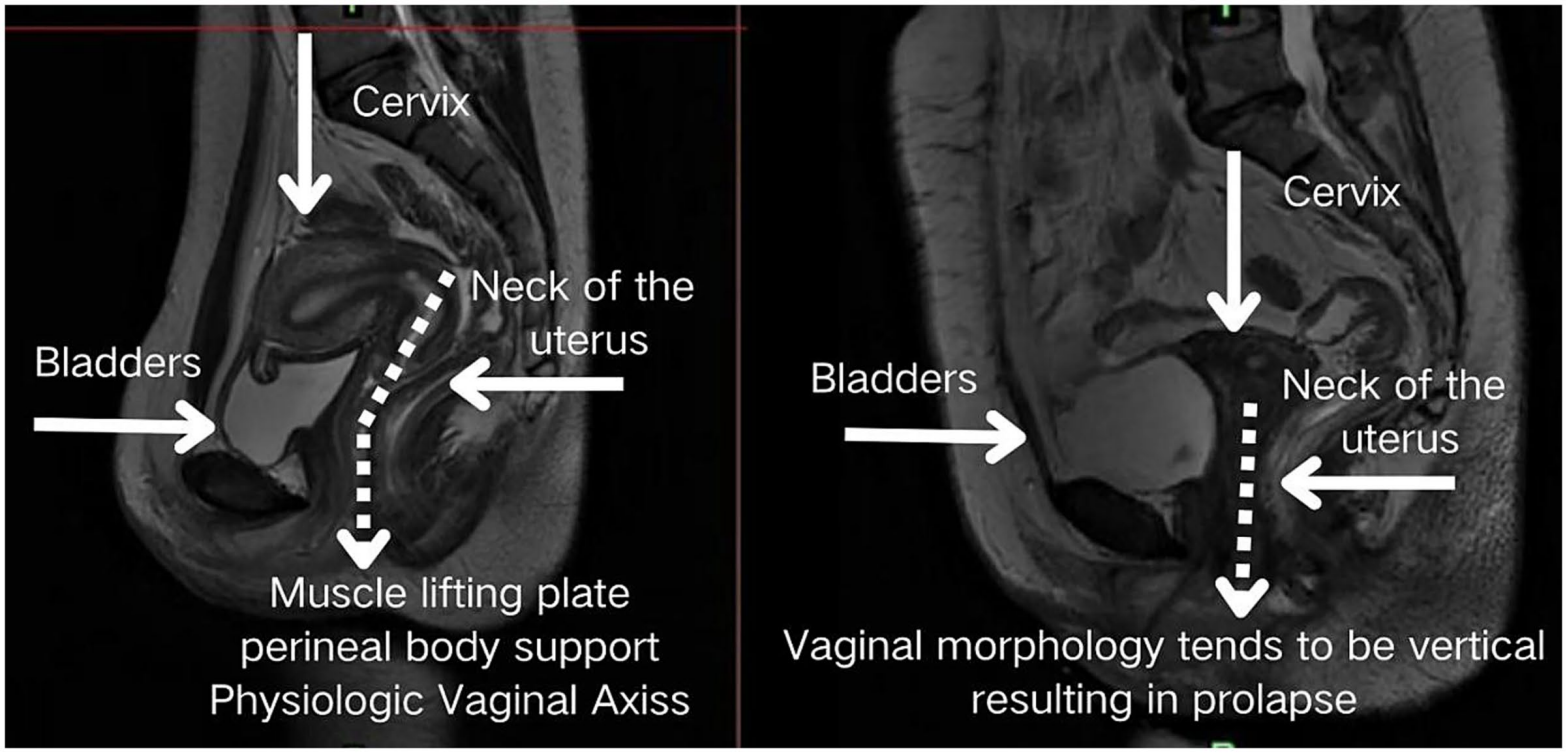
MRI validation of the piston-cylinder mechanism in a patient.

Structurally, the posterior fornix integrates the levator plate, pelvic floor ligaments, and perineal muscles (14) to form a dynamic fulcrum. This concave structure not only facilitates reproductive functions but also mechanically guides characteristic uterine displacement: when loaded, the uterus undergoes a “downward-backward” movement with concomitant anterior rotation at the fundus. This piston-cylinder-like mechanism—illustrated conceptually in Figure 3 and corroborated by patient MRI in Figure 4—redirects stress vectors posteriorly and establishes a protective mechanical coupling that resists uterine prolapse.

## Discussion

### Pelvic floor biomechanical mechanisms: from dynamic equilibrium to disruption

Our team previously conducted a biomechanical analysis study based on 20 subjects, which included 10 patients with pelvic organ prolapse (POP) and 10 healthy controls with preoperative and postoperative pairwise comparisons. Using dynamic magnetic resonance imaging (MRI) combined with finite element modeling techniques, we systematically analyzed the displacement vectors and stress distribution patterns of the pelvic organs in the state of increased abdominal pressure. The results showed that control subjects exhibited physiologically consistent posterior inferior displacement of the uterus and efficient transmission of mechanical stress along the sacrococcygeal axis, whereas patients in the POP group showed significant displacement vector abnormalities (*p* < 0.01) and a significantly greater angle of inclination of the caudal side of the anal raphe plate compared with the control group. Postoperative data showed a 15.87% reduction in the area of the anal raphe fissure in the horizontal plane and a 105.33% increase in the area of the perineal body in the coronal plane, indicating that the surgical intervention significantly improved the anatomy. Of particular importance, the reconstruction of the posterior vault effectively curbed the tendency of uterine prolapse from a biomechanical mechanism. These preliminary results support our proposed theoretical model and confirm the central role of restoring key anatomical structures and mechanical balance in the reconstruction of pelvic floor function.

Pelvic organ prolapse, a highly prevalent condition affecting 30–50% of parous women (1), originates in the failure of a dynamically regulated biomechanical system. This substantial clinical burden—exacerbated by persistent recurrence rates of 12–23% after conventional repair (2)—underscores the limitations of static anatomical reconstruction and emphasizes the need to investigate dynamic functional breakdown. Pelvic organ prolapse fundamentally arises from the failure of a precisely regulated biomechanical system maintained through three integrated mechanisms. First, the triangular support complex formed by the bladder, uterine cervix, and levator plate facilitates force distribution through coordinated actions: stress transmission via the posterior bladder wall (15), maintained uterine anteversion, and active levator plate contraction. The levator plate’s characteristic superoanterior contraction vector rapidly displaces the posterior vaginal wall, mechanically stabilizing the urethrovesical junction through bladder neck closure and functional urethral lengthening (16). The proposed triangular support complex consists of the posterior bladder wall (anterior vertex), the uterine cervix (superior vertex), and the levator plate (postero-inferior base). This configuration functions as a stable framework for force transmission. During increases in abdominal pressure, stress from the bladder is transmitted posteriorly to the cervix and inferiorly to the levator plate. Concurrently, the contraction of the levator plate generates a counteracting supero-anterior force, stabilizing the cervix and, by extension, the entire anterior compartment. The integrity of this dynamic triangle is therefore paramount in preventing a downward and anterior displacement of organs toward the vaginal introitus. Second, the posterior fornix serves as a pivotal fulcrum, directing characteristic posteroinferior uterine displacement and rotational motion to redistribute stresses toward the sacrococcygeal axis (17). Third, vaginal wall apposition at a critical 90° functional angle optimizes load transfer efficiency. Disruption of any component alters principal stress vectors toward the vaginal introitus, precipitating prolapse. These mechanistic insights establish a scientific framework for targeted pelvic floor reconstruction, emphasizing restoration of the levator plate complex, posterior fornix dynamics, and vaginal angulation.

The pathophysiology of pelvic floor dysfunction follows a well-defined biomechanical sequence originating from structural compromise. Critical to this process is perineal body shortening (30–50% reduction), which increases levator plate inclination by 15–25° (18), thereby redirecting pelvic stress vectors from the physiologic sacrococcygeal axis toward the vaginal introitus. Concurrent levator plate avulsions, particularly at the characteristic 3 and 9 o’clock positions, impair contractile efficiency by 40–60% (19), resulting in pathologic vaginal angulation (120 ± 10° versus normal 90°). These morphological alterations—including vaginal lumen dilation and loss of the functional folding angle—reduce stress transfer efficiency by 35–50% (20), severely compromising load-bearing capacity. The deterioration progresses through additional support system failures: loss of uterine anteversion exacerbates abnormal force distribution, while defects in the pubocervical fascia directly weaken anterior vaginal wall fixation (8). Together, these changes initiate a biomechanical vicious cycle: levator hiatus enlargement → vaginal axis deviation → organ descent → stress redistribution imbalance → clinically evident prolapse. This mechanistic understanding precisely identifies reparative targets, including restoration of perineal body dimensions, levator plate integrity, and vaginal angulation, providing an evidence-based foundation for surgical reconstruction.

Our findings on the critical biomechanical role of the posterior fornix and levator plate dynamics should be integrated with the static supportive function of pelvic ligaments, as emphasized in the Integral Theory, to comprehensively understand the mechanisms of pelvic floor support. The levator plate generates active dynamic forces, while the uterosacral, cardinal, broad, and round ligaments collectively form a passive structural support system. Serving as key anchoring points, they not only provide structural stability to the uterus, cervix, and vagina but also synergistically participate in shaping and transmitting force vectors within the pelvic floor system (21). These ligaments, composed mainly of collagen and elastic fibers, rely on microstructural integrity for maintaining pelvic organ stability. Once the collagen is disorganized or loosely structured, its mechanical properties will be significantly weakened, leading to abnormal force vector conduction and imbalance of stress distribution, and ultimately triggering pelvic organ prolapse (22).

The integrity of the uterosacral ligament complex is particularly critical for maintaining the spatial configuration of the vaginal-cervical axis. It guides the characteristic posteroinferior displacement of the uterus during increased abdominal pressure, thereby redirecting mechanical stress toward the sacrum and effectively reducing the load on the vaginal introitus (23). As one of the stiffest tissues in the pelvic floor, the ligament exhibits high stiffness and nonlinear viscoelastic behavior under low to medium strain rates, providing mechanical stability to the core supportive structures (22). In cases of weakened or injured pelvic floor muscles, the ligaments must compensatorily bear additional abdominal pressure to maintain organ position. If muscular dysfunction persists, prolonged stretching can lead to viscoelastic failure (e.g., creep and stress relaxation) and even microstructural damage in the ligaments, resulting increased organ displacement and progression of prolapse (24). Therefore, the synergistic interaction between ligaments and pelvic floor muscles under elevated intra-abdominal pressure is essential for protecting connective tissues from abnormal stress.

### Clinical significance

Contemporary management of pelvic organ prolapse is undergoing a transformative evolution, transitioning from traditional anatomical reconstruction to precision biomechanical restoration. This perspective coincides with DeLancey’s theory. He emphasized that traditional prolapse repair surgery often focuses narrowly on defects in a single compartment, while overlooking the fact that pelvic organ prolapse actually results from the abnormal distribution of intra-abdominal pressure across multiple compartments (25). Mounting clinical evidence exposes the limitations of conventional approaches, with long-term data demonstrating concerning recurrence rates (23.2% at 7 years post vaginal wall repair/sacral fixation) and high rates of *de novo* defects (81%) (26). This therapeutic impasse has catalyzed the development of innovative strategies targeting the pelvic floor’s dynamic equilibrium. Recent advances include 3D finite element-guided posterior fornix angle correction (maintaining 90–100°), which reduces apical recurrence by 41% (8.3% vs. 14.1%, *p* = 0.02) while improving sexual function scores (ΔPISQ-12 = +35%) (17). Concurrently, levator plate-external anal sphincter complex reconstruction demonstrates enhanced force-coupling efficiency and functional recovery (27). These biomechanically-informed interventions, validated through multimodal assessment (POP-Q, ICIQ-VS, dynamic MRI), now represent the standard of care per AJOG 2023 guidelines.

Contemporary classification systems recognize three biomechanical subtypes of pelvic floor dysfunction: anterior-predominant (posterior bladder wall stress abnormalities), apical-deficient (posterior fornix dysfunction), and mixed-type (multi-system compromise). This stratification enables targeted interventions: apical defects require posterior fornix angle restoration and force redirection; anterior defects demand bladder-vaginal space reconstruction; mixed cases need comprehensive repair. Dynamic reconstruction fundamentally shifts treatment goals from anatomical repositioning to active biomechanical regulation, demonstrating superior outcomes versus traditional approaches. Because of the measures to achieve mechanistically based interventions (28). The paradigm advances beyond morphological correction to functional restoration while enabling personalized treatment. Preventive measures like restrictive episiotomy (29) maintain mechanical equilibrium pre-pathology. While promising, some note potential oversimplification of biological variability, warranting stratified trials comparing approaches (Table 2).

**Table 2:** Comparison of concepts in pelvic floor repair procedures.

Core elements	Traditional static restoration	Dynamic biomechanical repair
Therapeutic target	Anatomical defect closure	Stress vector redirection
Mechanisms	Passive ligament support	Active muscle modulation + Angle control
Evaluation criteria	Simple anatomical repositioning (POP-Q staging)	Complex assessment (Anatomical + Functional + Imaging)
Typical procedure	Anterior and posterior vaginal wall repair	Posterior fornix plasty + levator plate reconstruction

## Limitations

The ideas and discussions presented in this article are based primarily on theoretical analysis, but the relevance and validity of these mechanisms in clinical practice have not been validated in large-scale clinical trials. In addition, there is individual heterogeneity in the biomechanical properties of pelvic floor structures (e.g., differences in age, delivery history, and physical fitness), and existing models have not yet fully encompassed the effects of these variables on mechanical balance. Future studies need to include more women with different physiologic and pathologic states to further validate the central role of dynamic mechanical balance and provide theoretical support for the development of new treatment strategies.

## Conclusion

The treatment of pelvic organ prolapse has undergone a fundamental paradigm shift from static anatomical reconstruction to dynamic biomechanical restoration, marking a transformative advancement in restoring functional pelvic support. Preliminary outcomes from our biomechanically-informed approach, which emphasizes posterior fornix reconstruction and levator plate rehabilitation, demonstrate its superior value over traditional repair: we observed a 90% anatomical success rate, alongside significant improvements in hiatal closure and functional recovery. This evolution recognizes that successful management requires not merely correcting anatomical defects but reestablishing the intricate balance of forces within the pelvic floor system. By addressing the root biomechanical dysfunctions through targeted interventions such as posterior fornix restoration and levator plate rehabilitation, contemporary approaches demonstrate superior clinical outcomes compared to traditional repairs. Looking forward, the field must prioritize the development of personalized treatment algorithms that account for individual biomechanical profiles while investigating strategies to sustain long-term pelvic floor equilibrium, ultimately achieving durable functional recovery for patients.

## Data Availability

The original contributions presented in the study are included in the article/Supplementary material, further inquiries can be directed to the corresponding author.
