# Supplementary material for: Dynamic biomechanical equilibrium in pelvic organ prolapse: from mechanistic insights to precision reconstruction
Source: Front Med (Lausanne). 2025 Oct 8;12:1637133. doi: 10.3389/fmed.2025.1637133 (PMC12542915; doi:10.3389/fmed.2025.1637133)
Supplement: Supplementary file 1 [file Supplementary_file_1.docx]

Patient 2D finite element simulation of pelvic floor deformation processes (double click to open)

Schematic animation of the pelvic floor deformation process (double click to open)
